# Olanzapine Plus Triple Antiemetic Therapy for the Prevention of Platinum-Based Delayed-Phase Chemotherapy-Induced Nausea and Vomiting: A Meta-Analysis

**DOI:** 10.3390/curroncol33010027

**Published:** 2026-01-04

**Authors:** Wenlin Gong, Hongxin Qie, Yuxiang Xu, Peiyuan Wang, Jinglin Gao, Mingxia Wang

**Affiliations:** Department of Clinical Pharmacology, The Fourth Hospital of Hebei Medical University, Shijiazhuang 050010, China

**Keywords:** CINV, olanzapine, platinum-based, chemotherapy, nausea, complete response

## Abstract

**Background:** Chemotherapy-induced nausea and vomiting (CINV) is a common treatment-related side effect that has a detrimental effect on the quality of life of patients with cancer and may lead to dose reductions or discontinuation of chemotherapy. This meta-analysis aims to explore the efficacy and safety of olanzapine plus triple antiemetic therapy for prevention of delayed-phase platinum-based CINV. **Methods:** Electronic databases (five English databases: (I) PubMed, (II) ScienceDirect, (III) The Cochrane Library, (IV) Scopus, and (V) EMBASE, and two Chinese databases: China National Knowledge Infrastructure and Wanfang Database) were searched for trials that evaluated the effectiveness and safety of olanzapine plus triple antiemetic in preventing platinum-based CINV. Efficacy was no nausea, complete control, and complete response (CR) rates in the acute, delayed, and overall phases after chemotherapy. Data were analyzed using the random effects model and fixed effects model. **Results:** A total of 18 trials involving 3110 patients were identified, including 9 controlled trials and 9 single-arm trials. The meta-analysis of nine studies, which showed significant heterogeneity (*p* = 0.002, I^2^ = 67%), demonstrated that the olanzapine (OLN) group had a significantly higher rate of delayed CR compared to the control group (OR: 2.33, 95% CI: 1.57–3.46, *p* < 0.00001). Compared with the Without OLN group, the With OLN group had a significant overall CR (OR: 2.18, 95% CI: 1.80–2.63, *p* < 0.00001, heterogeneity: *p* < 0.00001, I^2^ = 69%), and a significant acute CR (OR: 2.28, 95% CI: 1.45–3.58, *p* < 0.00001, heterogeneity: *p* = 0.04, I^2^ = 51%). The meta-analysis revealed that the With OLN group could significantly increase the risk of dry mouth compared to the Without OLN group (OR  =  2.60, 95% CI: 1.73–3.91). In terms of insomnia, the odds ratio for the With OLN group was significantly lower than that for the Without OLN group (OR = 0.60; 95% CI 0.41–0.89). **Conclusions:** The results of this meta-analysis provide robust evidence that adding olanzapine to standard triple therapy significantly improves the prevention of platinum-based delayed-phase CINV, a setting where current antiemetic regimens often prove suboptimal. However, it also increases the risk of certain adverse events, especially dry mouth. Clinical decisions should be made based on a thorough assessment of the therapeutic benefits and safety risks.

## Simple Summary

Chemotherapy-induced nausea and vomiting are common side effects of cancer treatment. Currently, the standard preventive regimen is known as “triple therapy,” yet its effectiveness remains suboptimal for some patients, particularly in managing delayed-phase chemotherapy-induced nausea and vomiting. Recent studies have shown that adding low-dose olanzapine to form a “quadruple therapy” can significantly enhance delayed-phase antiemetic efficacy. This new approach has been increasingly incorporated into international clinical guidelines. Furthermore, this successful case has opened new research avenues in “drug repurposing” and “multi-target synergistic therapy.” Future investigations are expected to extend beyond traditional antiemetics, exploring agents that act on multiple neural receptor pathways, with a growing emphasis on personalized and precision medicine strategies.

## 1. Introduction

Chemotherapy-induced nausea and vomiting (CINV) is a common and frightening accompanying symptom during chemotherapy, which can affect appetite and mood in mild cases, interrupt chemotherapy in severe cases, and significantly shorten disease-free survival (DFS) and overall survival (OS) [1]. Studies have shown that without standardized antiemetic prophylaxis, the incidence of vomiting exceeds 90% in patients receiving highly emetogenic chemotherapy (HEC) [2].

Although all HEC regimens require potent antiemetic regimens, platinum-based drugs (especially cisplatin) stand out due to their extremely significant and persistent delayed vomiting characteristics. Platinum-based drugs, widely used in chemotherapy, are a class of metal complexes that inhibit cancer cell growth by interfering with DNA replication, including cisplatin, carboplatin, and oxaliplatin, among others. Their efficacy is broad-spectrum and not limited to specific cancer types, making them fundamental components of many first-line standard chemotherapy regimens. For example, in lung cancer treatment, the TP regimen (paclitaxel plus carboplatin) and EP regimen (etoposide plus cisplatin) are widely used, while in ovarian cancer, the TC regimen (paclitaxel plus carboplatin) is also a standard option [3]. Given their extensive clinical application, effective management of CINV associated with these drugs is particularly important. Compared to other highly emetogenic regimens, such as the AC regimen (doxorubicin combined with cyclophosphamide), platinum-based drugs carry a higher risk of delayed vomiting, necessitating special attention in clinical management [4].

The complex pathophysiological mechanism of CINV has directly spurred the development and application of modern antiemetic drugs, with the core principle being the combined blockade of different neurotransmitter pathways. In clinical practice, antiemetic drugs are mainly divided into five categories based on their mechanism of action: 5-HT3 receptor antagonists, neurokinin-1 (NK1) receptor antagonists, dopamine (D_2_) receptor antagonists, antihistamines and anticholinergic drugs, and glucocorticoids [5]. In highly emetic drugs, such as platinum-based chemotherapy (especially cisplatin), the combination of 5-HT3 receptor antagonists, NK-1 receptor antagonists, and dexamethasone is routinely recommended by domestic and foreign guidelines [6].

Previous phase III clinical studies reported that the effective rate of the three-drug combination strategy for controlling highly emetic drugs (including patients who did not experience vomiting and those who did not require rescue treatment) was 50% to 70% [7]. In a study by Takanori Miyoshi et al. [8], involving 267 patients, two antiemetic regimens based on triple therapy were compared. The analysis focused on patients receiving at least moderately emetogenic chemotherapy. The total complete response rates were 97.8% in the acute phase and 83.1% in the delayed phase. Therefore, the efficacy of triple therapy in the acute phase is better than that in the delayed phase. Although the triple therapy includes NK-1 receptor antagonists, which are effective in the delayed phase, it has been found in practice that the protective effect of a single dose on the day of chemotherapy may gradually weaken in the following days. It can be seen from this that the triple therapy has a poor preventive effect on delayed nausea and vomiting. In contrast, 5-HT3 receptor antagonists targeting the acute phase have a direct and powerful effect, so the control rate of the triple therapy in the acute phase (usually >90%) is higher than that in the delayed phase [9].

The control of CINV associated with platinum-based chemotherapy regimens, particularly the prevention of delayed-phase symptoms (2–5 days after chemotherapy), remains a significant clinical challenge [6,10]. Although the triple antiemetic regimen, comprising an NK-1 receptor antagonist, a 5-HT3 receptor antagonist, and dexamethasone, is considered the standard of care, its protective efficacy during the delayed phase remains suboptimal in a considerable proportion of patients. Olanzapine, a multi-receptor-targeting agent with affinity for dopaminergic, histaminergic, and serotonergic (including 5-HT_3_) receptors, has emerged as a promising adjunct for “full-cycle” antiemetic protection. It effectively mitigates emetic triggers in the acute phase, with a pronounced effect on nausea control. Its broad receptor coverage and sustained pharmacokinetic profile also provide continued antiemetic activity during the delayed phase [11]. Although sedation is a recognized side effect [12], growing evidence suggests that adding olanzapine to the standard triple therapy may further improve control of delayed CINV. Therefore, the aim of this study is to evaluate, via a prospective randomized controlled trial, the preventive effect of a quadruple regimen—combining low-dose olanzapine with a standard triple antiemetic therapy—in patients receiving highly emetogenic platinum-based chemotherapy. This investigation is based on existing evidence supporting the efficacy of olanzapine for chemotherapy-induced nausea and vomiting (CINV).

## 2. Methods

### 2.1. Search Strategy and Selection Criteria

#### 2.1.1. Search Strategy

The following five databases were used to search for relevant articles: (I) PubMed, (II) ScienceDirect, (III) The Cochrane Library, (IV) Scopus, (V) EMBASE, and two Chinese databases (China National Knowledge Infrastructure and Wanfang Database). “Olanzapine AND platinum-based” and “Olanzapine AND Cisplatin”, “Olanzapine AND Chemotherapy-induced nausea and vomiting”, “platinum-based” were used as key terms to illustrate the search strategy. This inquiry focused on randomized controlled trials (RCTs) published before September 2025. The database search included additional filters: “clinical trial”, “full text”, and “species: human”. We reviewed all potentially eligible studies without imposing restrictions on language or primary outcomes. Furthermore, a manual search of reference lists from relevant reviews was conducted.

#### 2.1.2. Selection Criteria

The research questions and eligibility criteria for the systematic review conformed to the PICOS (participants, interventions, comparators, outcomes, and study design) approach. Studies meeting the following criteria were considered for inclusion:

(1) Participants: patients were ≥18 years old who were diagnosed with cancer and naïve to chemotherapy;
(2) Outcomes: complete response (CR, defined as no emesis and no use of rescue medication) in the overall (0 to 120 h), acute (0 to 24 h), and delayed (24 to 120 h) phases; the proportion of patients who have complete control and no nausea in the phases above;
(3) Study design: experimental group (With olanzapine: olanzapine plus triple antiemetic therapy) versus control group (without olanzapine: triple antiemetic therapy);
(4) Chemotherapy regimen: platinum-based.

### 2.2. Data Extraction and Quality Assessment

W.G. and H.Q. screened the literature, extracted data independently using a standardized table, and cross-checked the information. The table includes relevant information, such as the author’s name, publication time, area, platinum category, olanzapine dosage, sample size, gender, intervention measures, and outcomes (Table 1 and Table 2). The two reviewers independently performed the data extraction and retrieved study details from the articles using a predefined questionnaire. Discrepancies in study selection or data extraction between reviewers were resolved by consultation with a third reviewer. Meta-analysis was performed using Review Manager 5.4 software. The quality of each included randomized controlled trial (RCT) was assessed using version 5.4.0 of the Cochrane Risk of Bias tool. This evaluation covered the following domains: random sequence generation, allocation concealment, blinding of participants and personnel, blinding of outcome assessment, incomplete outcome data, selective reporting, and other bias. The risk of bias in each domain was judged and categorized as “low risk”, “high risk”, and “unclear risk” [13]. Low risk: The relevant biases are clearly addressed, and the methods used are appropriate. High risk: The method used is clearly non-random. Unclear risk: The authors did not provide any relevant information; such details are missing.

### 2.3. Statistical Analyses

The treatment effects for dichotomous outcomes were estimated in pairwise comparisons, with results reported as odds ratios (ORs) and their 95% confidence intervals (CIs). All meta-analyses were conducted using Review Manager software (version 5.4). Regarding the merger effect quantity, *p* ≤ 0.05 was considered statistically significant. I^2^ statistics were used to measure statistical heterogeneity. Based on the observed heterogeneity, with I^2^ values not exceeding 25%, a fixed-effect model employing the Mantel-Haenszel method was applied. For analyses demonstrating higher heterogeneity (I^2^ > 25%), a random-effects model was utilized [14]. A heterogeneity test value of ≥25% was considered to indicate significant heterogeneity, prompting the use of sensitivity analysis. To assess the robustness of our findings, we performed a sensitivity analysis using the Leave-One-Out method.

### 2.4. PRISMA Guidelines

This meta-analysis was conducted in accordance with the Preferred Reporting Items for Systematic Reviews and Meta-Analyses (PRISMA) guidelines (Registration Number: CRD420251236352) (Supplementary File S1).

**Table 1:** Characteristics of included studies with control groups.

			Platinum Category	Study Design		Sample Sizes		Gender (n): M, F		Age (Years)		Efficacy Endpoint	Type of Cancer
Author/Year	Olanzapine Dosage	Platinum- Based	Dosage	With OLN	Without OLN	With OLN	Without OLN	With OLN	Without OLN	With OLN	Without OLN		With OLN, Without OLN
Hironobu Hashimoto 2020 [15]	5 mg	Cisplatin	≥70 mg/m^2^ <70 mg/m^2^ One course of treatment	OLN + APR/FOS + PALO + DEX	Placebo + APR/FOS + PALO + DEX	354	351	237, 118	234, 117	Median 65	Median 66	CR/CC/TC	Head and neck 33, 25 Lung 179, 183 Esophageal 75, 79 Gastric 20, 19 Gynecological 34, 34 Urological 3, 1 Other 11, 10
Satoshi Koyama 2023 [16]	5 mg/10 mg	Cisplatin	100 mg/m^2^, One course of treatment	OLN + APR + PALO + DEX	APR + PALO + DEX	31	78	25, 6	71, 7	Mean 61.6	Mean 64.2	CR	Head and neck cancer 31, 78
Jiali Gao 2022 [17]	5 mg	Cisplatin	25 mg/m^2^/d, 3 day	OLN + APR + TRO + DEX	APR + TRO + DEX	59	61	32, 27	31, 30	Mean 60.39	Mean 58.11	CR	Lung cancer 31, 31 Others 28, 30
YuanyuanZhao 2022 [18]	5 mg	Cisplatin	3-day total dose ≥ 75 mg/m^2^	OLN + FOS + OND + DEX	PAL + FOS + OND + DEX	175	174	137, 38	134, 40	Median 60	Median 58	CR/NN	Lung 126, 126 Head and neck 24, 21 Other 25, 27
Masakazu Abe 2015 [19]	5 mg	Cisplatin	<50 mg/m^2^ ≥50 mg/m^2^	OLN + APR + PALO/GRA + DEX	APR + PALO/GRA + DEX	50	50	0, 50	0, 50	Mean 53	Mean 53	CR/CC/TC/NN/NV/NRT	Uterine cervical cancer 23, 23 Uterine corpus cancer 22, 22 Uterine carcinosarcoma 2, 2 Ovarian cancer 2, 2 Vaginal cancer 1
Yan Zhang 2024 [20]	5 mg	Cisplatin	25 mg/m^2^/d, 3 day	OLN + FOS/APR + GRA/TRO + DEX	FOS/APR + GRA/TRO + DEX	23	23	14, 9	15, 8	Mean 57.5	Mean 59	CR	Lung cancer 13, 15 Other 10, 8
Lulu Zhang 2025 [21]	5 mg	Cisplatin	20 mg/m^2^ /day, 5 day	OLN + APR + TRO + DEX	Placebo + APR + TRO + DEX	77	77	77, 0	77, 0	Median 28	Median 28	CR/NN/TC	Testicular tumor 65 Mediastinal tumor 8 Other 4
Naoki Inui 2024 [22]	5 mg	Carboplatin	AUC ≥ 5 mg/mL/min	OLN + PALO/GRA + APR + DEX	Placebo + APR + PALO/GRA + DEX	175	180	139, 36	140, 40	Median 72	Median 72	CR/CC/TC/NN	Lung adenocarcinoma 82, 95 Squamous cell lung carcinoma 33, 33 Small cell lung cancer 38, 33 Others 12, 19
Vikas Ostwal 2024 [23]	10 mg	Oxaliplatin, Carboplatin	Oxaliplatin not given; Carboplatin: AUC ≥ 5 mg/mL/min	OLN + PALO + APR + DEX	PALO + APR + DEX	274	270	180/102	179/99	Median 51	Median 50	CR/NN/NV	Colorectal 165, 159 Gastric or gastroesophageal 22, 22 Non-small cell lung carcinoma 26, 29 Biliary tract carcinoma 31, 30 Biliary tract carcinoma 24, 24 Others 12, 14

OLN: olanzapine; PALO: palonosetron; APR: aprepitant; FOS: fosaprepitant; DEX: dexamethasone; TRO: tropisetron; OND: ondansetron; GRA: granisetron; CR: complete response (no vomiting, no rescue, and any nausea); CC: complete control (no vomiting, no rescue, and nausea grade 0 or 1); TC: total control (no vomiting, no rescue, and nausea grade 0); NN: no nausea; NV: no vomiting; NRT: no rescue therapy; AUC: area under curve.

**Table 2:** Characteristics of the included single-arm experimental group studies.

Author/Year	Country	Olanzapine Dosage	Platinum Type	Cisplatin-Dosage	Study Design	Sample Sizes	Gender (n): M, F	Age (Years)	Efficacy Endpoint	Type of Cancer
Hiroko Minatogawa 2024 [24]	Japan	5 mg	Cisplatin	≥50 mg/m^2^	OLN + PALO + NK1 + DEX	139	95, 44	Median age 63	CR	Esophageal 56 Head and neck 32 Lung 25 Gastric 10 Others 16
Hiroko Minatogawa 2024(2) [24]	Japan	5 mg	Cisplatin	≥50 mg/m^2^	OLN + PALO + NK1 + DEX	139	97, 42	Median 64	CR	Esophageal 53 Head and neck 37 Lung 28 Gastric 6 Others 15
Hirotoshi Iihara 2020 [25]	Japan	5 mg	Carboplatin	≥4 mg/mL/min	OLN + APR + GRN + DEX	57	0, 57	Median58	CR	Ovarian cancer 26 Cervical cancer 7 Endometrial cancer 21 Others 3
Jun Wang 2022 [26]	China	5 mg	Cisplatin	100 mg/m^2^	OLN + APRT + TRO + DEX	75	58, 17	Mean 46	CR	Nasopharyngeal carcinoma
Jun Wang 2022(2) [26]	China	10 mg	Cisplatin	100 mg/m^2^	OLN + APRT + TRO + DEX	75	58, 17	Mean 46	CR	Nasopharyngeal carcinoma
Kazuhisa Nakashima 2017 [27]	Japan	5 mg	Cisplatin	75 mg/m^2^	OLN + APR + PALO + DEX	30	27, 3	Median 64	CR	Lung cancer 40 Others 64
Kazuki Tanaka 2019 [28]	Japan	5 mg	Carboplatin	≥6 mg/mL/min	OLN + 5HT3 + APRT/FOS + DEX	33	29, 4	Median 75	CR	Non-squamous NSCLC 33
Masakazu Abe 2016 [29]	Japan	5 mg	Cisplatin	≥50 mg/m^2^	OLN + APR + PALO + DEX	40	0, 40	Median 57	CR	Cervical cancer 20 Endometrial cancer 19 Vulval cancer
Junichi Nishimura 2021 [30]	Japan	5 mg	Oxaliplatin	85 mg/m^2^	OLN + APR + PALO + DEX	40	23, 17	Median 60	CR	Colorectal cancer 40

OLN: olanzapine; PALO: palonosetron; APR: aprepitant; FOS: fosaprepitant; DEX: dexamethasone; TRO: tropisetron; CR: complete response (no vomiting, no rescue, and any nausea); NK1: neurokinin-1 receptor antagonists (NK-1 RA); NSCLC: non-small-cell carcinoma.

## 3. Results

### 3.1. Study Selection

**Figure 1:**
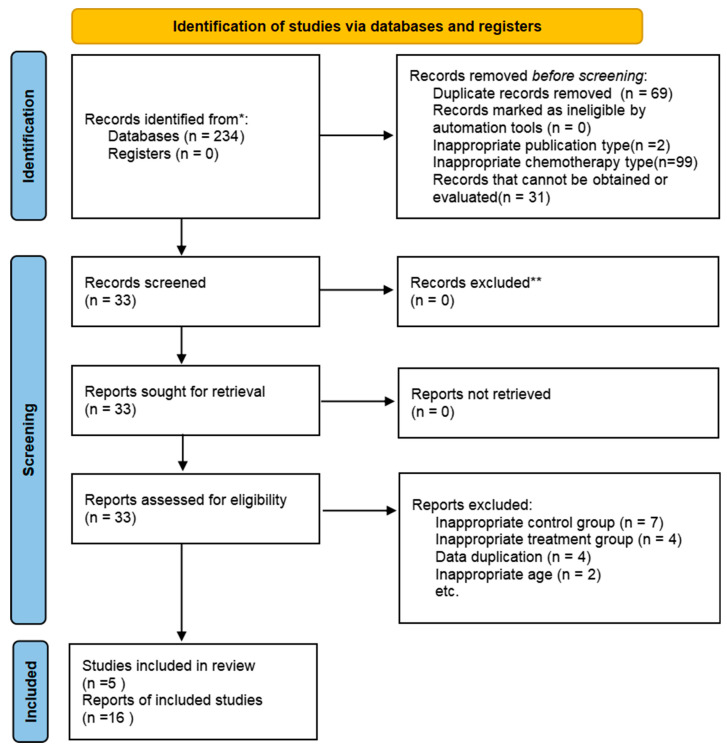
Flow diagram of the trial search and selection process.

In total, 234 studies were retrieved by electronic and manual search methods. A total of 69 of them were excluded because of duplication. A total of 33 articles were selected and closely scrutinized for eligibility. A total of 17 articles were further excluded for the following reasons: (1) inappropriate control group (*n* = 7); (2) inappropriate treatment group (*n* = 4); (3) data duplication (*n* = 4); (4) inappropriate age (*n* = 2); 16 articles assessing 3110 patients (With olanzapine group, 1218 patients; Without olanzapine group, 1264 patients; the single-arm experiment involved 628 individuals) were ultimately included in the analysis (Figure 1). Among them, the experimental group and control group of the two articles were used as single-arm experiments. Therefore, although only 16 articles [15,16,17,18,19,20,21,22,23,24,25,26,27,28,29,30] were included in this article, it included 18 trials.

### 3.2. Study Characteristics and Quality

**Figure 2:**
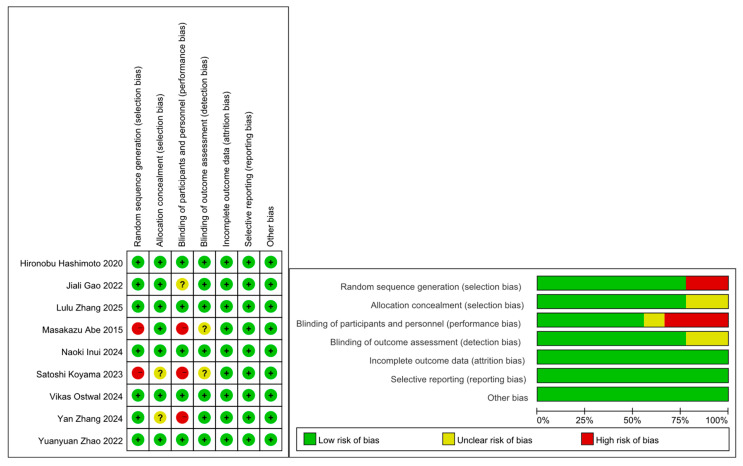
Quality assessment of included studies. Hironobu Hashimoto 2020 [15]; Satoshi Koyama 2023 [16]; Jiali Gao 2022 [17]; YuanyuanZhao 2022 [18]; Masakazu Abe 2015 [19]; Yan Zhang 2024 [20]; Lulu Zhang 2025 [21]; Naoki Inui 2024 [22]; Vikas Ostwal 2024 [23].

This article ultimately included 16 articles [15,16,17,18,19,20,21,22,23,24,25,26,27,28,29,30], 9 articles [15,16,17,18,19,20,21,22,23] with control group experiments, and 7 articles [24,25,26,27,28,29,30] (9 experiments) as single-arm studies included in this study. We evaluated the eligibility criteria of the nine identified studies [15,16,17,18,19,20,21,22,23] (including experimental and control groups) using the Cochrane Collaboration tool. The quality analysis of all studies is shown in Figure 2. Among the randomized controlled trials included in this study, five studies had a relatively low risk of bias in their randomization process. However, the remaining four RCTs [16,17,19,20] raised concerns about bias arising from insufficient information on concealment and participant allocation. Random sequence generation was inadequate in two trials [16,19]. And allocation concealment was adequately described in two trials [16,17,18,19,20]. Overall, five RCTs [15,18,21,22,23] had a low overall risk of bias, whereas the remaining four RCTs [16,17,19,20] raised concerns of potential bias. The following information was summarized in Table 1 and Table 2: author and year of publication, area, study design, number of assigned patients, chemotherapy, and outcomes.

### 3.3. Complete Response and Other Efficacy Outcomes

#### 3.3.1. Complete Response Rate

##### CR with Controlled Clinical Trial

We defined the absence of vomiting and the lack of reliance on emergency medications as criteria for a complete response. We evaluated the effectiveness of CR based on overall, acute, and delayed responses.

**Figure 3:**
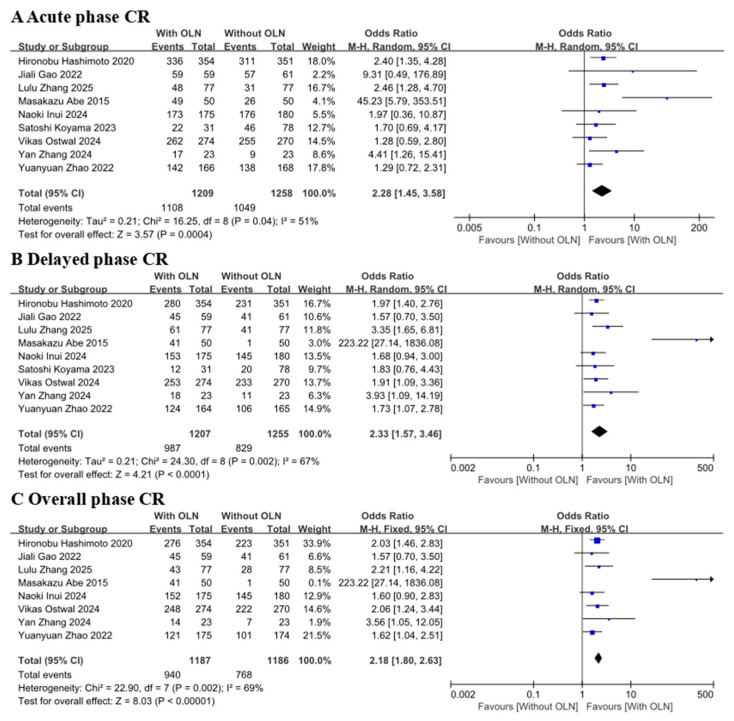
Forest plot for the complete response of CINV. With OLN group versus Without OLN group for patients with cancer. Abbreviations: 95% CI: 95% confidence intervals; M-H: Mantel-Haenszel; With OLN: With olanzapine; Without OLN: Without olanzapine; CR: complete response (no emetic episodes and no use of rescue medication). Hironobu Hashimoto 2020 [15]; Satoshi Koyama 2023 [16]; Jiali Gao 2022 [17]; YuanyuanZhao 2022 [18]; Masakazu Abe 2015 [19]; Yan Zhang 2024 [20]; Lulu Zhang 2025 [21]; Naoki Inui 2024 [22]; Vikas Ostwal 2024 [23].

In nine studies [15,16,17,18,19,20,21,22,23] examining the delayed CR (heterogeneity *p* = 0.002, I^2^ = 67%), compared with the Without OLN group, the With OLN group demonstrated a superior response (OR: 2.33, 95% CI: 1.57–3.46, *p* < 0.0001; Figure 3, Group B). Although an OR > 1 typically indicates increased risk, in this context, the “risk” actually refers to the probability of a complete response (CR). Therefore, an OR > 1 signifies a greater chance of CR occurring in the experimental group. This result suggests that olanzapine can help prevent the occurrence of CINV. This delayed-phase effect was consistent across both the acute and overall phases.

Eight studies [15,17,18,19,20,21,22,23] were included in the overall CR analysis (heterogeneity: *p* = 0.002, I^2^ = 69%). Compared with the control group, the experimental group had a significant CR (OR: 2.18, 95% CI: 1.80–2.63, *p* < 0.00001; Figure 3 Group C). Additionally, nine studies [15,16,17,18,19,20,21,22,23] were included in the acute CR analysis (heterogeneity: *p* = 0.04, I^2^ = 51%). Compared with the control group, the experimental group had a much higher CR (OR: 2.28, 95% CI: 1.45–3.58, *p* = 0.0004; Figure 3 Group A). The *p*-values are all less than 0.05, indicating statistical significance in this analysis. I^2^ is greater than 25%, indicating significant heterogeneity. After removing Masakazu Abe 2015 [19], I^2^ decreased to 0, and the heterogeneity *p*-values for acute, delayed, and total phases were 0.45, 0.76, and 0.86, respectively. Masakazu Abe 2015 [19] is the reason for the high heterogeneity in this group.

To evaluate the robustness of the results, a sensitivity analysis was performed using the Leave-One-Out method, given that the heterogeneity (I^2^) exceeded 25% across all three periods. Each study was removed sequentially, and the aggregate effect size was recalculated to examine its influence on the overall findings. The results indicated that after excluding the study by Masakazu Abe 2015 [19], the heterogeneity (I^2^) decreased to zero. However, the pooled effect size did not change significantly, suggesting that this particular study did not exert a directional impact on the overall results.

##### CR of Single Arm

**Figure 4:**
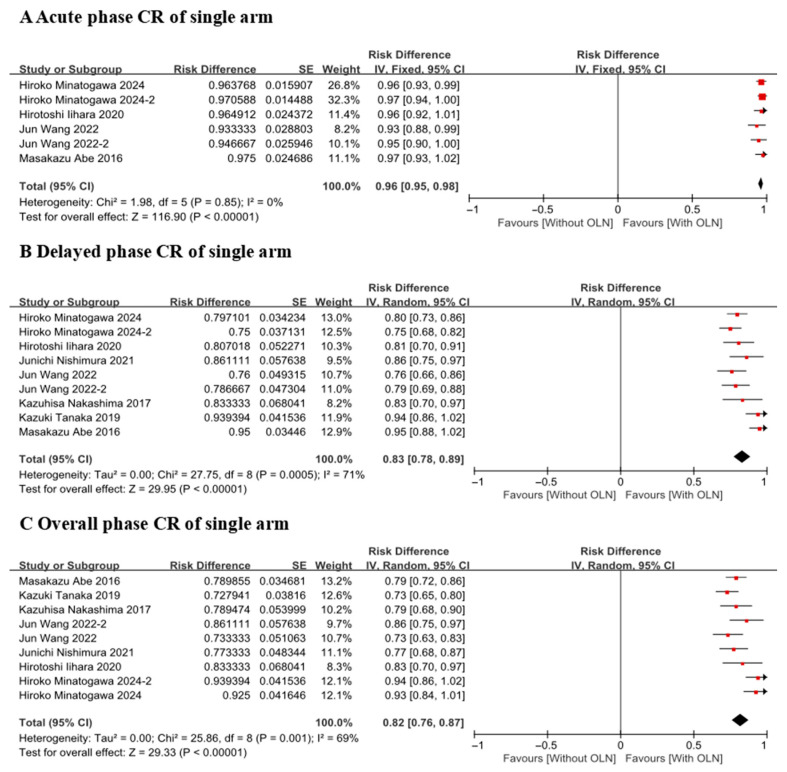
Forest plot for the complete response of CINV. With OLN group. Abbreviations: 95% CI: 95% confidence intervals; SE: standard error; With OLN: With olanzapine; CR: complete response (no emetic episodes and no use of rescue medication).Hiroko Minatogawa 2024 [24]; Hiroko Minatogawa 2024(2) [24]; Hirotoshi Iihara 2020 [25]; Jun Wang 2022 [26]; Jun Wang 2022(2) [26]; Kazuhisa Nakashima 2017 [27]; Kazuki Tanaka 2019 [28]; Masakazu Abe 2016 [29]; Junichi Nishimura 2021 [30].

Since the I^2^ statistic for the acute phase was less than 25%, a fixed-effects model was applied. In contrast, as the I^2^ values for both the delayed and total phases exceeded 25%, random-effects models were employed for these analyses. The CR of platinum-based chemotherapy-induced delayed phase [24,25,26,27,28,29,30] nausea and vomiting treated by olanzapine in adult patients was 0.83 (95% CI = 0.78–0.89, I^2^ = 71%, *p* = 0.0005) (Figure 4, Group B). CR of the acute phase [24,25,26,29] was the highest (RD (Risk Difference) = 0.96, 95% CI = 0.95–0.98, I^2^ = 0%, *p* = 0.85) (Figure 4, Group A). CR of overall phase [24,25,26,27,28,29,30] was the lowest (RD = 0.82, 95% CI = 0.76–0.87, I^2^ = 69%, *p* = 0.001) (Figure 4, Group C).

Given that the heterogeneity (I^2^) exceeded 25% in both the delay and total phase, a sensitivity analysis was performed using the Leave-One-Out method to assess the robustness of the model. This involved iteratively removing each study, recalculating the pooled effect size, and I^2^ statistic. The results demonstrated that neither the combined effect size nor the I^2^ value changed substantially following the exclusion of any individual study. This indicates that the model exhibits high stability with the current dataset.

#### 3.3.2. No Nausea Rate or No Vomiting Rate

**Figure 5:**
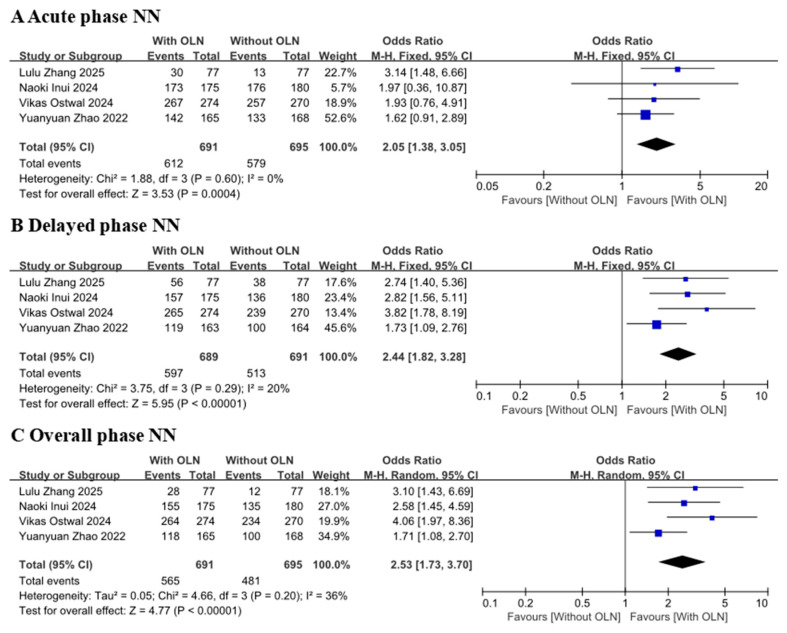
Forest plot for the no nausea rate, or no vomiting rate of CINV. With OLN group versus Without OLN group for patients with cancer. Abbreviations: 95% CI: 95% confidence intervals; M-H: Mantel-Haenszel; With OLN: With olanzapine; Without OLN: Without olanzapine; NN: No nausea. YuanyuanZhao 2022 [18]; Lulu Zhang 2025 [21]; Naoki Inui 2024 [22]; Vikas Ostwal 2024 [23].

Pooled results of the 4 studies [18,21,22,23] showed that the incidence of no nausea in the control group (OR: 2.05, 95% CI: 1.38–3.05, *p* = 0.0004; Figure 5 Group A) was significantly lower than that of the experimental group (heterogeneity: *p* = 0.60, I^2^ = 0%) in the acute phase. Four studies [18,21,22,23] evaluated the delayed phase (heterogeneity: *p* = 0.29, I^2^ = 20%), and it was found that in this summary analysis, the incidence of no vomiting was significantly increased in the experimental group compared to the control group (OR: 2.44, 95% CI: 1.82–3.28, *p* < 0.00001; Figure 5, Group B). For the overall phase [18,21,22,23], the results showed that the experimental group had a much higher effect than the control group (*p* < 0.00001, OR:2.53, 95% CI:1.73–3.70; Figure 5, Group C). Due to the high heterogeneity (*p* = 0.20, I^2^ = 36%), a randomized controlled model was selected.

Given the substantial heterogeneity observed in the overall phase NN (I^2^ = 36%), we performed a Leave-One-Out sensitivity analysis. Each study was sequentially removed, and the model was recomputed. The analysis revealed that the heterogeneity notably decreased when the studies by Vikas Ostwal 2024 [23] and Yuanyuan Zhao 2022 [18] were excluded, with I^2^ values dropping to 10% and 0%, respectively.

### 3.4. Safety

#### 3.4.1. AEs Included in Controlled Clinical Trial Studies

**Figure 6:**
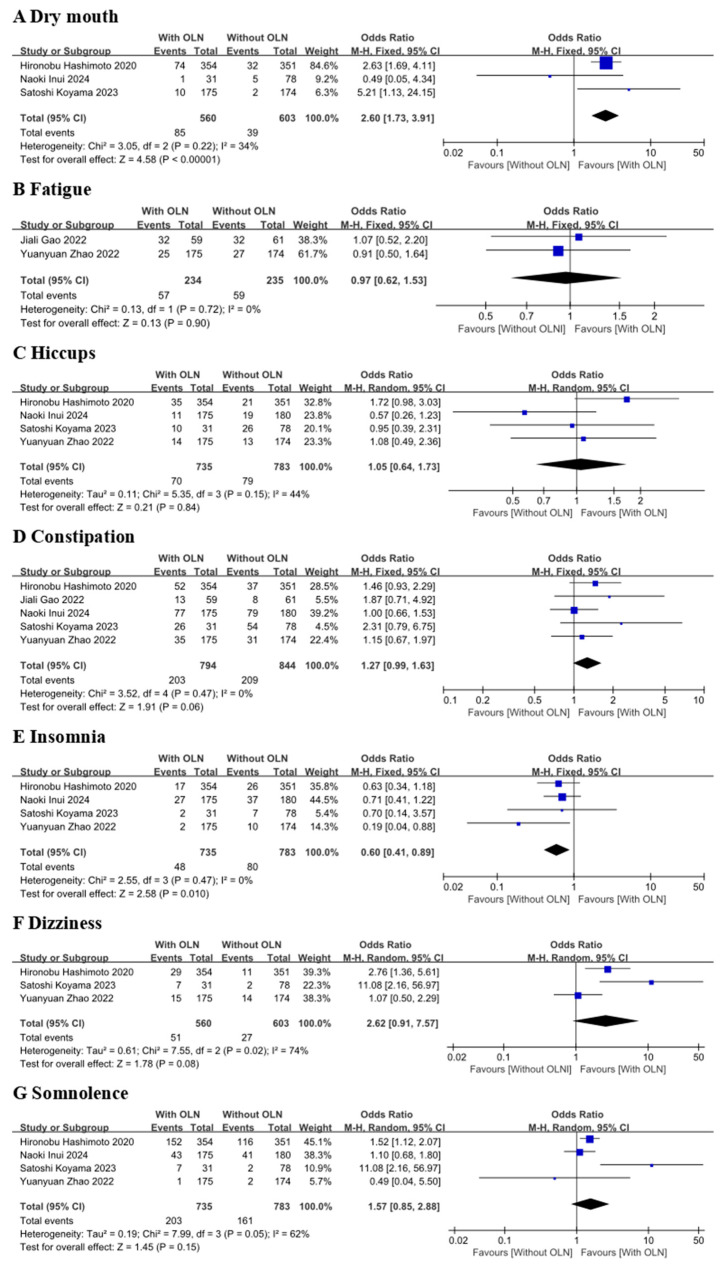
Forest plot of adverse events: With OLN group versus Without OLN group for patients with cancer. Abbreviations: 95% CI: 95% confidence intervals; M-H: Mantel-Haenszel; With OLN: With Olanzapine; Without OLN: Without Olanzapine. Hironobu Hashimoto 2020 [15]; Satoshi Koyama 2023 [16]; YuanyuanZhao 2022 [18]; Naoki Inui 2024 [22]; Jiali Gao 2022 [17].

Seven adverse events (AEs) of dizziness, constipation, hiccups, somnolence, dry mouth, fatigue, and insomnia were included in the quantitative analysis of this meta-analysis (see Figure 6). Hiccups, dizziness, and somnolence were found to be heterogeneous. Therefore, a random model was used (Figure 6, Groups C, F, and G). For the rest of the AEs, no significant heterogeneity was detected, and a fixed model (Figure 6, Groups A, B, D, and E) was used in this review. According to five studies, there was a significant difference in the overall incidence and frequency of treatment-emergent adverse events (TEAEs) occurring during treatment between the control group and the experimental group.

The meta-analysis revealed that the experimental group, compared to the control group, did not show a significantly lower risk for developing dizziness [15,16,18] (OR 2.62, 95% CI 0.91–7.57) (Figure 6, Group F). For hiccups [15,16,18,22] (*p* = 0.15, I^2^ = 44%) and somnolence [15,16,18,22] (*p* = 0.05, I^2^ = 62%), the OR value was greater than 1 (hiccup: OR = 1.05, 95% CI: 0.64–1.73, *p* = 0.84 Figure 6 Group C; somnolence: OR = 1.57, 95% CI: 0.85–2.88, *p* = 0.15 Figure 6 Group G).

Meta-analysis demonstrated that the use of olanzapine increased the risk of dry mouth [15,16,22] rates more than the control group (OR = 2.60; 95% CI, 1.73–3.91; *p* < 0.00001) (Figure 6, Group A). There was high statistical heterogeneity for these factors (*p* = 0.22, I^2^ = 34%). For other adverse reactions with significant statistical differences observed, the OR value of fatigue [17,18] (*p* = 0.72, I^2^ = 0%) was lower than 1 (OR = 0.97, 95% CI: 0.62–1.53, *p* = 0.90) (Figure 6, Group B). The incidence of fatigue is not statistically significant. Focusing on combination therapies, in terms of constipation [15,16,17,18,22], the odds ratio for the OLN group was significantly higher than that for the control group (OR 1.27; 95% CI 0.99–1.63) (Figure 6 Group D). The experimental group did not significantly increase the risk of insomnia [15,16,18,22] compared to the control group (OR  =  0.60, 95% CI: 0.41–0.89) (Figure 6, Group E).

In this meta-analysis, it was found that the *p*-values of dry mouth and insomnia were lower than 0.05, indicating statistical significance. The incidence of insomnia was higher in the group without olanzapine, while the incidence of dry mouth was higher in the experimental group. After excluding the two studies that used a 10 mg dose of olanzapine, only the result for somnolence changed, showing a statistically significant increase in risk (OR = 1.38, 95% CI: 1.06–1.80, *p* = 0.02). Heterogeneity was eliminated (I^2^ = 0%, *p* = 0.39), and the confidence interval no longer crossed the line of no effect and has statistical significance.

Figure 6 indicates that significant heterogeneity (I^2^ > 25%) was present in the Somnolence, Hiccups, Dry mouth, and Dizziness groups, which prompted a Leave-One-Out sensitivity analysis. For the Dizziness group, heterogeneity remained high (I^2^ > 25%), and the confidence interval continued to cross the null value (OR = 1) regardless of which study was excluded. In contrast, for the Somnolence group, exclusion of the study by Satoshi Koyama 2023 [16] reduced heterogeneity to zero, and the confidence interval no longer crosses invalid values, indicating this study had a directional influence on the results. For the Hiccups group, heterogeneity dropped to zero upon excluding either Hironobu Hashimoto 2020 [15] or Naoki Inui 2024 [22], although the confidence interval still crossed invalid values. Finally, for the Dry mouth group, excluding Naoki Inui 2024 [22] eliminated heterogeneity (I^2^ = 0%) without altering the confidence interval, suggesting no directional impact from this study.

#### 3.4.2. AEs Included in the Single-Arm Experimental Group Study

AEs were reported in nine studies (Table 3). All adverse reactions observed in the single-arm trial are listed in Table 3. Some common AEs of concern were analyzed. Combining all 9 single-arm studies [24,25,26,27,28,29,30] reporting safety data, the incidence rate of constipation [24,25,26,27,28,29,30] was 56.0% (RD = 0.51, 95% CI: 0.27–0.75; I^2^ = 98%, *p* < 0.00001), while the incidence rate of hiccups [24,25,26,27,28] (reported by 7 studies) was 45.9% (RD = 0.38; 95% CI: 0.14–0.62; I^2^ = 98%, *p* < 0.00001). Furthermore, the pooled estimates of incidence rate of somnolence (reported by all 9 trials [24,25,26,27,28,29,30]) and insomnia (reported by 3 trials [24,25]) were 56.2% (RD = 0.51; 95% CI: 0.37–0.66; I^2^ = 94%, *p* < 0.00001) and 32.5% (RD = 0.32; 95% CI: 0.27–0.37; I^2^ = 0%, *p* = 0.73). Finally, the incidence rate of Dry mouth (reported by 3 trials [27,28]) was 63.0% (RD = 0.63; 95% CI: 0.58–0.68; I^2^ = 0%, *p* = 0.93). The *p* < 0.05 values for all five subgroups are statistically significant. Regarding other adverse reactions, see Table 3. If the I^2^ statistic exceeds 25% for an adverse reaction, a random-effects model is selected; otherwise, a fixed-effects model is employed.

A Leave-One-Out sensitivity analysis was performed on groups exhibiting high heterogeneity (I^2^ > 25%). The results showed no substantial changes in either heterogeneity or the pooled effect size for any group after the sequential removal of individual studies, confirming the robustness of the overall conclusions.

## 4. Discussion

We conducted a meta-analysis of studies reviewed evaluating the efficacy and safety of olanzapine aimed at improving platinum-based CINV control. This study included a total of 16 articles (18 experiments) and found that the percentage of patients with CR during the delayed phase was significantly higher in the experimental group compared with the control group (81.8% vs. 66.1%), as were the percentages in the overall (79.2% vs. 64.8%) and acute phases (91.6% vs. 83.4%). The experimental group was also superior to the control group during the delayed and overall phases (83.6% vs. 69.2%) for secondary efficacy end points of no nausea.

Our study revealed that the addition of olanzapine to the triple combination therapy effectively reduced the incidence of platinum-based CINV. Even without NK1 receptor antagonists in the treatment plan, the use of olanzapine could still have a good preventive effect on delayed nausea [31,32]. Due to poor safety, it is recommended to consider using olanzapine in patients with refractory delayed nausea.

In previous studies, it was found that olanzapine was equally effective in preventing chemotherapy-induced nausea and vomiting at oral doses of 5 mg and 10 mg per day for 1–4 days, but the incidence of adverse events was higher at the 5 mg dose than at the 10 mg dose [33]. Ithimakin et al. [33] reported that the 5 mg olanzapine group had a higher incidence of adverse reactions such as constipation, headache, fatigue, muscle spasms, and diarrhea; the 10 mg group experienced more frequent reactions such as hiccups and bloating. In conclusion, the overall incidence of adverse reactions in the 5 mg group is still relatively high. Historically, a 5 mg dose has been used in many antiemetic regimens; however, emerging evidence now supports the efficacy of even lower doses. A recent phase III randomized controlled trial demonstrated that a 2.5 mg dose of olanzapine is non-inferior to the standard 10 mg dose when combined with triplet antiemetic therapy for highly emetogenic chemotherapy (HEC) [34]. A comparison of the 2.5 mg and 10 mg olanzapine doses in combination with triplet therapy for HEC is therefore warranted.

Olanzapine significantly improves the complete response (CR) rate in patients, thus helping to ensure treatment tolerance and compliance. Severe, uncontrolled CINV is a primary reason leading patients to refuse or prematurely terminate effective chemotherapy regimens. By markedly increasing the CR rate—particularly in cases of delayed CINV, which is the most difficult to control—olanzapine directly enables patients to complete their planned chemotherapy cycles without compromising anticancer efficacy due to intolerable side effects. This translates to a higher likelihood of tumor remission and improved survival.

Furthermore, olanzapine enhances quality of life and psychological well-being by freeing patients from the persistent fear and anticipatory anxiety associated with post-chemotherapy nausea and vomiting. This allows patients to achieve meaningful rest and recovery during treatment intervals, rather than suffering through days of distress.

This research performed an extensive evaluation of the effectiveness of olanzapine by employing various outcome measures. In this meta-analysis, non-statistically significant and ineffective adverse reactions were excluded. The incidence of insomnia was higher in the group without olanzapine, while the incidence of dry mouth was higher in the olanzapine treatment group. There was no statistically significant correlation between the research factors and the results. While the olanzapine (OLN) group demonstrated superior therapeutic efficacy, the study failed to establish a significant safety difference between the two groups.

After excluding two studies with a 10 mg dose of olanzapine, no significant changes were observed in other outcome measures except for somnolence. The result revealed a statistically significant increase in the risk of somnolence in the experimental group (OR = 1.38, 95% CI: 1.06–1.80, *p* = 0.02). Heterogeneity for this outcome was eliminated (I^2^ = 0%, *p* = 0.39). Somnolence is an adverse reaction associated with the sedative effect of olanzapine, whereas insomnia represents an opposite type of adverse event. Overall, these results suggest that the use of low-dose olanzapine is associated with a higher probability of adverse reactions in the treatment group.

A comparative analysis of adverse reactions between the controlled trial and the single-arm study revealed a considerable overlap in the types observed. However, due to the limited number of available studies and the consequent high risk of bias, only adverse reactions reported in three or more literature sources were discussed here. In the single-arm study, dry mouth was the most common adverse reaction (63%), followed by somnolence (56.2%), and insomnia remained the least frequent (32.5%). In single-arm trials, two studies reported appetite loss as an adverse reaction. In fact, increased appetite is a commonly recognized adverse effect of olanzapine. However, this discrepancy can be explained by a counteracting mechanism: while olanzapine alone may stimulate appetite, chemotherapy-induced nausea and vomiting act as strong appetite suppressants. Consequently, the net observed effect in these patients was a reduction in appetite. In the analysis of AEs from controlled clinical trials, those that lacked statistical significance and ineffective drug-related causality were excluded. The results showed a higher incidence of insomnia in the non-olanzapine group and a higher incidence of dry mouth in the olanzapine group. These findings were consistent with the adverse reaction profile observed in the single-arm studies. Despite a higher incidence of adverse reactions associated with olanzapine, all observed symptoms were controllable. Effective and targeted management strategies for common reactions include dry mouth, which is managed by frequent, small-volume water intake; diarrhea, which can be effectively controlled with agents such as montmorillonite powder; and drowsiness, which is addressed by maintaining a regular sleep schedule and incorporating short daytime naps.

Several limitations in this article include, firstly, we cannot disregard the potential impact of different ethnic groups on the experiment. In addition, a potential limitation of this study is the lack of a stratified discussion of gender in receiving treatment plans. Being female and young, two predisposing risk factors for CINV, further increases their susceptibility [35]. It is necessary to conduct larger sample size studies in the future, discussing age, region, and ethnicity separately to confirm our findings. Finally, “beyond-delayed” nausea and vomiting (defined as occurring beyond 120 h) has gained increasing attention due to its greater resistance to control compared to acute or delayed phases. However, this study did not evaluate outcomes related to this “beyond-delayed” period, and its efficacy in this context remains unaddressed.

## 5. Conclusions

In conclusion, this meta-analysis establishes that the addition of olanzapine to triple antiemetic therapy confers a significant benefit in preventing platinum-based CINV, effectively addressing the critical unmet need in the management of delayed-phase symptoms. Although olanzapine use is associated with an increased incidence of dry mouth, this side effect remains manageable.

## Figures and Tables

**Table 3:** Forest plot characteristics: single-arm studies.

AEs	E	n	N	Prop. (%)	Heterogeneity (*p*, I^2^)	RD	95% CI
Constipation	9	350	625	56.0	*p* < 0.00001, I^2^ = 98%	0.51	0.27–0.75
Somnolence	9	351	625	56.2	*p* < 0.00001, I^2^ = 94%	0.51	0.37–0.66
Hiccups	7	250	545	45.9	*p* < 0.00001, I^2^ = 98%	0.38	0.14–0.62
Dry mouth	3	209	332	63.0	*p* = 0.93, I^2^ = 0%	0.63	0.58–0.68
Insomnia	3	108	332	32.5	*p* = 0.73, I^2^ = 0%	0.32	0.27–0.37
Dizziness	2	24	97	24.7	*p* < 0.00001, I^2^ = 95%	0.12	0.07–0.18
Anxious	2	54	275	19.6	*p* = 0.32, I^2^ = 0%	0.19	0.15–0.24
Fatigue	2	175	275	63.6	*p* = 0.10, I^2^ = 62%	0.64	0.55–0.73
Appetite loss	2	188	275	68.4	*p* = 0.001, I^2^ = 90%	0.69	0.51–0.86
Anorexia	2	32	73	43.8	*p* = 0.09, I^2^ = 65%	0.44	0.25–0.63
Headache	2	86	275	31.3	*p* = 0.003, I^2^ = 92%	0.31	0.12–0.50
Diarrhea	2	17	97	17.5	*p* < 0.0001, I^2^ = 94%	0.06	0.02–0.11
Nephrotoxicity	1	3	33	9.1	/	/	/
Hepatotoxicity	1	15	33	45.5	/	/	/
Thrombocytopenia	1	20	33	60.6	/	/	/
Anemia	1	24	33	72.7	/	/	/
Neutropenia	1	28	33	84.8	/	/	/
Leukopenia	1	24	33	72.7	/	/	/

AEs: Adverse events; E: Number of experiments included; n: Number of adverse reactions; N: Total number of people; Prop. (%): Proportion of participants who reported adverse events; RD: Risk difference; /: The limited number of studies precluded the creation of a forest plot.

## Data Availability

No new data were created or analyzed in this study. Data sharing is not applicable to this article.

## Supplementary Materials

The following supporting information can be downloaded at: https://www.mdpi.com/article/10.3390/curroncol33010027/s1. File S1. PRISMA 2020 Checklist.
